# Balloon assisted gastrostomy tube placement

**DOI:** 10.1007/s00261-025-04962-4

**Published:** 2025-04-30

**Authors:** Norbert Kuc, Ariel Felman, Ilan Small, Jacob Cynamon, Arash Gohari

**Affiliations:** 1 Montefiore Medical Center, The Bronx, USA; 2 Albert Einstein College of Medicine, The Bronx, USA; 3 Columbia University Irving Medical Center, New York, USA; 4 St. John’s Riverside Hospital, Yonkers, USA

**Keywords:** Gastrostomy, Interventional, PEG, Balloon, BAG, G-tube

## Abstract

**Purpose:**

To compare the safety and efficacy of balloon-assisted gastrostomy (BAG) placement to the conventional serial dilation technique.

**Methods:**

This study is an IRB-approved retrospective review of all percutaneous gastrostomy tubes placed by an interventional radiology department at a single institution between 2012 and 2021. There were 476 patients identified (average age 63, 44% female): 385 in the serial dilation group and 91 in the balloon assisted gastrostomy (BAG) group. Patient demographic, procedure, and radiological data were reviewed in the medical record to determine procedure success, procedure/fluoroscopy time, and tube failures. Gastrostomy tube failure was defined as tube leak, clogging, or dislodgement. Adverse events were classified as per Society of Interventional Radiology guidelines. Statistical analysis was performed using Fisher’s exact test, student’s t-test, and Mann-Whitney U-test as appropriate.

**Results:**

Gastrostomy tubes were successfully placed in 97.7% (377/385) of patients undergoing the serial dilation technique and 100% (91/91) of patients undergoing the BAG placement technique. BAG tube placement was associated with a 2.5 min decrease (47%) in average fluoroscopy time ( *p*  = 0.0002, CI: 3.76 to 1.20). Total procedure time was reduced by an average of 17.2 min (22%) ( *p*  = 0.0006, CI: 26.9 to 7.4). BAG was also associated with an 11% reduction in all cause gastrostomy tube failure ( *p*  = 0.0399). There were no statistically significant differences in the adverse event rates or median days to tube failure. Material costs were $178.32 higher in the BAG group.

**Conclusion:**

BAG catheter placement can be performed safely and effectively, and is associated with reduced fluoroscopy time, procedure time, and overall failure rate compared to the serial dilation technique.

**Graphical abstract:**

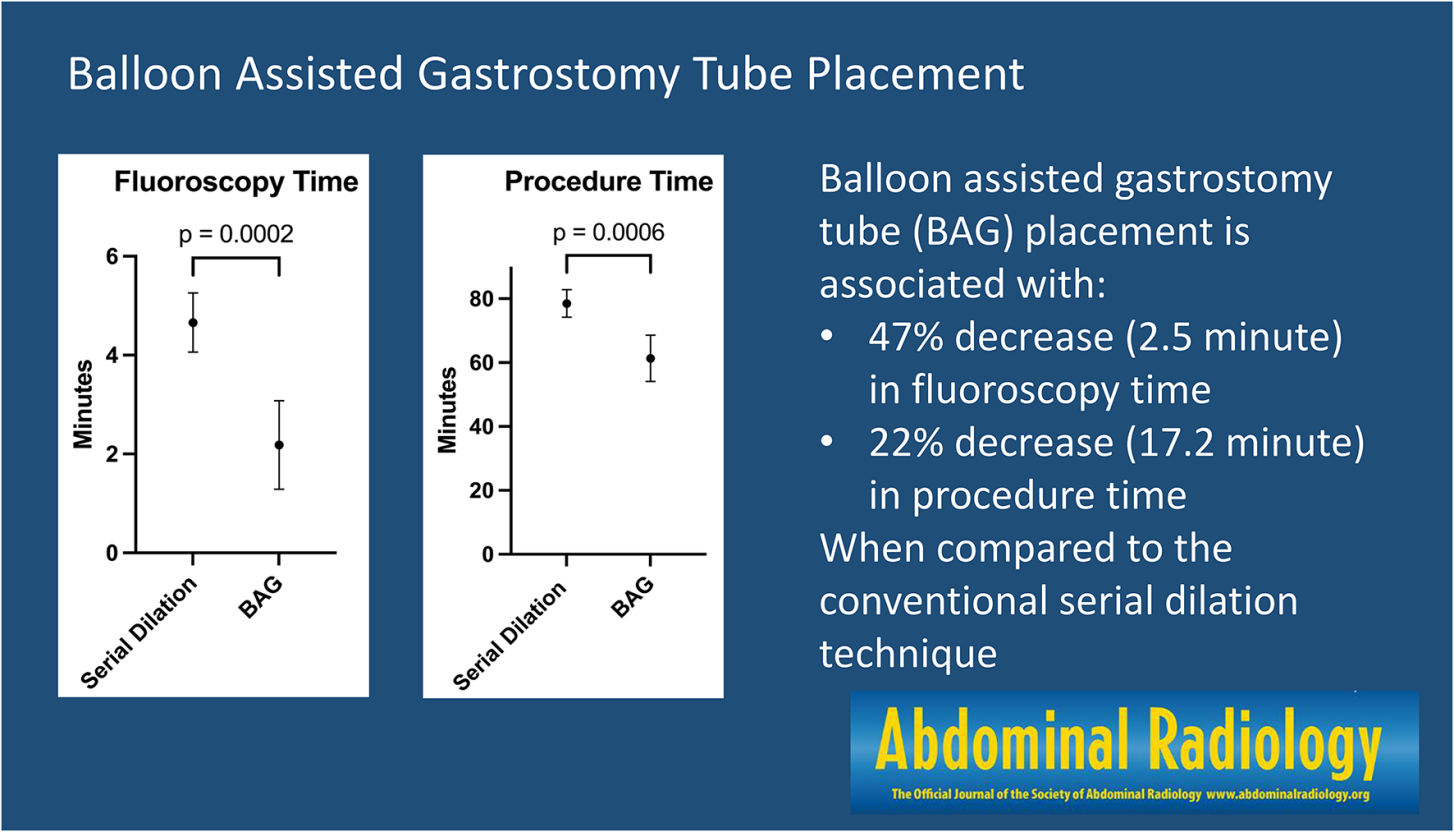

**Supplementary Information:**

The online version contains supplementary material available at 10.1007/s00261-025-04962-4.

## Introduction

Gastrostomy tubes are placed in patients as a well-tolerated means of providing enteral nutrition for those unable to receive oral nutrition and is a commonly performed procedure. Common indications for placement of gastrostomy tubes include patients on ventilators, head and neck malignancies, gastric decompression, and neurologic disorders [1]. Gastrostomy tubes are placed by interventional radiology techniques, as well as surgically and endoscopically [2]. Gastrostomy tubes placed under fluoroscopic guidance by interventional radiologists may avoid the limitations of other placement techniques, such as tumor seeding, complications of invasive surgeries, and operating room/anesthesia utilization [3]. It was previously suggested that percutaneous image guided gastrostomy tube placement is preferred placement method due to its high level of procedure success and relatively low cost [4]. Image guided gastrotomy tube placement has become a staple procedure in any hospital-based interventional radiology department, yet there is still room for further optimization and refinement of the technique to increase throughput, safety, and reduce failures. Studies have demonstrated that gastrostomy tube placement is a significant risk factor in 30-day hospital readmission, and reducing failure rates may lead to a reduction in healthcare resource utilization [5].

Image guided gastrostomy catheter placement is traditionally performed using the serial dilation technique. This technique is characterized by inserting progressively larger dilators percutaneously into the stomach to size up the tract before placement of the gastrostomy tube [1]. An alternative technique involves dilating the tract using an angioplasty balloon. This dilation technique may be performed in a single step, which may potentially decrease procedure time and radiation exposure. This technique has been successfully described in the literature and was suggested to provide benefits over traditional serial dilation, however small sample sizes have limited the statistical significance and generalizability of prior studies [6–8]. Following the introduction and perceived benefits of the balloon assisted gastrostomy (BAG) placement technique, operators in this study transitioned to primarily performing the BAG technique.

The purpose of this study is to compare the BAG and serial dilation gastrostomy tube placement techniques to determine outcomes of the procedure in a single institution after switching to a primarily BAG approach.

## Methods

This is an IRB-approved retrospective review of gastrostomy tubes placed percutaneously by BAG or serial dilation in an interventional radiology department at a single institution between 2012 and 2021 at a major metropolitan academic center. Patients were identified via the Montage (Nuance mPower, Burlington, MA) database. Inclusion criteria included all patients who underwent initial percutaneous gastrostomy tube placement with a minimum 90-day follow-up. Charts were manually reviewed for age, sex, body mass index (BMI), procedure time, active fluoroscopy time, indication, adverse events, tube failure, and time to tube failure. Tube failure was defined as tube leak, clogging, or dislodgement. Procedure time was calculated by determining the total amount of time between when the primary operator was marked as “arrived” in the angiography suite in nursing documentation, and the end of procedure documentation time in the electronic medical record. Adverse events were classified according to the Society of Interventional Radiology guidelines [9].

Gastrostomy tubes were placed by 6 experienced operators accompanied by a rotation of trainees at 3 different hospitals within the same institution. All operators performed the serial dilation technique before transitioning primarily to a BAG technique during the timeframe of the study.

### Statistical analysis

Categorical variables were analyzed using Fisher’s exact test, quantitative variables with expected equal variances were analyzed using the student’s t-test, and quantitative variable with expected unequal variances were analyzed using the Mann-Whitney U-test. A p-value of < 0.05 was considered statistically significant. Statistical analysis was performed in GraphPad Prism 10 (GraphPad Software, Boston, MA).

#### Serial dilation gastrostomy tube placement technique

Prior to the procedure, cross sectional imaging was reviewed to determine if a safe window was present between the liver and transverse colon for placement of a percutaneous gastrostomy tube. If cross sectional imaging was not available, barium was administered 12 h before the procedure for opacification of the colon to aid visualization in the angiography suite.

**Fig. 1:**
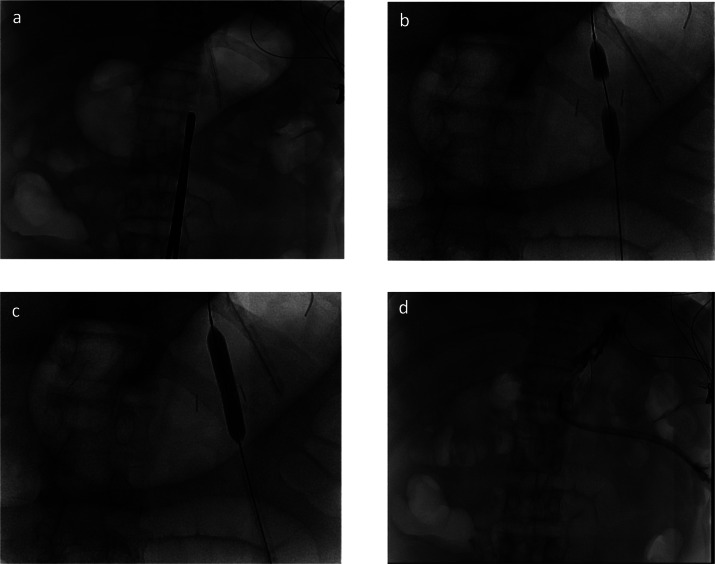
Representative fluoroscopic images of percutaneous radiologic gastrostomy placement: (**a**) Stomach insufflated via indwelling NG tube with radiopaque clamp marking proposed puncture site. (**b**) Balloon angioplasty catheter inserted between 2 gastropexy anchors, partially inflated. (**c**) Balloon catheter fully inflated. (**d**) Water-soluble contrast injected through gastrostomy tube confirming correct placement.

A nasogastric (NG) tube was used to inflate the stomach to place it against the anterior abdominal wall. Under fluoroscopic guidance, the stomach air bubble was visualized, and a clamp was placed over the anterior abdominal wall to mark the site of entry (Fig. 1A). T-fasteners were used to fasten the stomach to the anterior abdominal wall and iodine contrast was injected into the stomach to confirm positioning. Once access to the stomach was confirmed, a stiff wire was advanced into the stomach. The puncture site was then dilated over the wire using serial dilation with 8 and 12 French dilators, followed by final dilation up to the gastrostomy tube size (usually 18Fr). A 22Fr peel away sheath was then inserted over the wire into the gastrostomy site, then the gastrostomy tube was loaded on the wire behind the sheath. The gastrostomy tube was then advanced into the stomach as the sheath was peeled.

#### Balloon assisted gastrostomy (BAG) placement technique

The BAG technique uses the same initial steps as the serial dilation method to obtain access and wire placement into the stomach. An 8–9 mm Conquest (Becton Dickenson, Franklin Lakes, NJ) or Mustang (Boston Scientific, Marlborough, MA) PTA angioplasty balloon was then preloaded inside the lumen of the gastrostomy tube from the rear and advanced until the balloon emerged fully from the tip of the gastrostomy tube. Next, the angioplasty balloon and gastrostomy tube were loaded onto the wire with intraluminal stomach access and advanced as a unit into the stomach, until a small portion of the proximal balloon was external to the patient. The balloon was then inflated creating a soft tissue waist (Fig. 1B). Once the waist was visualized, the balloon was fully inflated until the tract was dilated (Fig. 1C). The angioplasty balloon was then slowly deflated while forward pressure was applied to the gastrostomy tube, and the gastrostomy tube and balloon were advanced as a unit into the lumen of the stomach. The angioplasty balloon was then fully deflated and removed from the stomach, and the gastrostomy catheter retention balloon was inflated and intraluminal stomach positioning confirmed using contrast and fluoroscopy (Fig. 1D).

## Results

There were 476 patients identified (average age 63, 44% female): 385 in the serial dilation group and 91 in the BAG group (Table 1). There was no significant difference in demographics, procedure success rate, or adverse event rates between the groups. Procedure time was significantly shorter in the BAG group (BAG: 61.3 ± 30.4 min, serial dilation: 78.5 ± 38.9 min, *p* = 0.0006), with a mean difference of 17.2 min (95% confidence interval: 26.9 to 7.4). Fluoroscopy time was significantly shorter in the BAG group (BAG: 2.18 ± 4.30 min, serial dilation: 4.63 ± 5.83 min, *p* = 0.0002), with a mean difference of 2.5 min (95% confidence interval: 3.76 to 1.20). All cause tube failure was higher in the serial dilation group vs. the BAG group (BAG: 15.4% vs. serial: 26.5%, *p* = 0.0399). There were no statistically significant differences between the individual causes of tube failures (Table 2).

**Table 1:** Patient demographics

Variable	Serial Dilation (*n* = 385)	BAG (*n* = 91)	*p*-value
**% Female**	43.9% (*n* = 169)	42.9% (*n* = 39)	0.950
**BMI ± SD**	25.7 ± 7.42	26.4 ± 7.86	0.424
**Age ± SD**	66.8 ± 15.2	68.7 ± 11.9	0.269
**Indication for procedure**			
Neurological Impairment	40.8% (*n* = 157)	40.7% (*n* = 37)	> 0.999
Ventilator Dependent	20.3% (*n* = 78)	38.5% (*n* = 35)	0.0005
Head and Neck Malignancy	17.9% (*n* = 69)	7.7% (*n* = 7)	0.0165
Obstruction	8.1% (*n* = 31)	4.4% (*n* = 4)	0.272
Other	13.0% (*n* = 50)	8.8% (*n* = 8)	0.372

**Table 2:** Outcomes of balloon assisted gastrostomy (BAG) vs. serial dilation gastrostomy catheter placement

Variable	Serial Dilation (*n* = 385)	BAG (*n* = 91)	*p*-value
**Procedure Success**	97.7% (*n* = 377)	100% (*n* = 91)	0.363
**Mean Procedure Time (minutes) ± SD**	78.5 ± 38.9	61.3 ± 30.4	0.0006 [CI: 26.9 to 7.4].
**Mean Fluoroscopy Time (minutes) ± SD**	4.63 ± 5.83	2.18 ± 4.30	0.0002 [CI: 3.76 to 1.20].
**All-Cause Tube Failure**	26.5% (*n* = 100)	15.4% (*n* = 14)	0.0399
Tube Dislodgement	49.0% (*n* = 49)	57.0% (*n* = 8)	-
Tube Leak	6.0% (*n* = 6)	0.0% (*n* = 0)	-
Tube Clogged	45.0% (*n* = 45)	43.0% (*n* = 6)	-
**Median Days to Failure (IQR)**	44 (123)	39 (79)	0.988
**Overall Adverse Events**	2.1% (*n* = 8)	3.2% (*n* = 3)	0.439
Hemorrhage	0.5% (*n* = 2)	2.2% (*n* = 2)	-
Infection	1.0% (*n* = 4)	0	-
Pneumoperitoneum	0.2% (*n* = 1)	0	-
Other	0.2% (*n* = 1)	1.1% (*n* = 1)	-
**Major (SIR Grade 3 or higher) Adverse Event Incidence (% of total AE)**	3 (38%)	0	0.182

There were significantly more patients receiving gastrostomy tubes via the BAG technique for a ventilator dependent indication compared to serial dilation (BAG: 38.5% vs. serial: 20.3%, *p* = 0.0005). Head and neck malignancy had a higher association with the serial dilation compared to BAG (BAG: 7.7% vs. serial: 17.9%, *p* = 0.0165) (Table 2).

Material costs for the dilation portion of the procedure amounted to $116.96 for the serial dilation method and $295.28 for the BAG method, amounting to a $178.32 difference. For the serial dilation method this cost included the serial dilators and peel away sheath. For BAG method, cost included the price of the angioplasty balloon and inflation device. These numbers excluded the cost of the gastrostomy balloon kit, which was the same between both methods.

A total of 8 procedure related adverse events (AE) were identified in the serial dilation group (Table 2). One patient was found to have significant pneumoperitoneum after the procedure, with no intervention required (SIR grade 1 AE). There were two incidents of post procedure bleeding, with one patient experiencing persistent gastrostomy tube site bleeding which resolved without significant intervention by discharge (SIR grade 1 AE). The second patient experienced severe hypotension after gastrostomy placement, and a CTA demonstrated hemoperitoneum and extravasation from the left gastric artery. An attempt was made to embolize the bleed however the patient expired during the embolization procedure (SIR grade 5 AE). There were 4 procedure related infections in the serial dilation group. Two patients experienced gastrostomy site cellulitis which resolved with treatment (SIR grade 2 AE). One patient experienced infection requiring hospital admission and IV antibiotic therapy (SIR grade 3 AE), and another patient developed an abscess at the gastrostomy insertion site, requiring prolonged admission (SIR grade 3 AE). There was one intra-procedural adverse event, where a patient experienced bradycardia which resolved with atropine administration (SIR grade 2 AE).

In the BAG group, there were a total of 3 procedure related adverse events. One patient was found to have a gastrointestinal bleed after gastrostomy tube placement which was treated with packed red blood cell transfusion and subsequently resolved (SIR grade 2 AE). Another patient experienced gastrostomy tube site bleeding which warranted a packed red blood cell transfusion before resolving (SIR grade 2 AE). There was one instance of prolonged pain after gastrostomy tube placement which required treatment with opioids (SIR grade 2 AE).

## Discussion

The present study introduces a large cohort of gastrostomy tube placements via the BAG technique. The results demonstrate a statistically significant reduction in fluoroscopy time and procedure time when using the BAG technique versus the serial dilation technique. BAG procedure times were reduced by over 17 min, amounting to a 22% reduction. Additionally, patients were exposed to an average of 47% less fluoroscopy time, amounting to less radiation for the patient and the operators in the room. Reduced procedure and fluoroscopy time allows for greater patient throughput and potential cost savings for this commonly performed procedure.

Studies have demonstrated that gastrostomy tube placement is a significant risk factor in 30-day hospital readmission [5]. Modifying procedure techniques to reduce failure rates may prevent hospital readmission and lower healthcare resource utilization. This study demonstrates an overall reduction in tube failure rate in the BAG group compared to the serial dilation group which was statistically significant and not detected on prior studies [6–8]. The all-cause failure rate was found to be about 11% lower with BAG placement compared to serial dilation placement. There were no differences detected between the subgroups of gastrostomy tube failure causes. Small subgroup sample sizes may have limited the study’s power to detect statistically significant differences in individual categories of tube failure causes. Alternatively, the BAG technique may be associated with a small but equal reduction among all individual causes of tube failures for which this study was only powered to detect in the all-cause failure analysis. Future studies with larger BAG cohorts or pooled meta-analyses may be able to improve sensitivity for this important metric.

Although the mechanism for reduced all-cause failure cannot be determined by this study, BAG may offer a less traumatic method of gastrostomy tract dilation, as the action of dilating is transferred from the operator forcibly inserting a stiffened catheter into the tract, to a more compliant angioplasty balloon. BAG may also offer better opposition of the anterior wall of the stomach to the anterior abdominal wall, as during the inflation process a “waist” is visualized (Fig. 1B), holding the soft tissues to be dilated in close opposition. This contrasts with the serial dilation technique, in which the action of passing a dilator percutaneously pushes apart the anterior abdominal wall and stomach wall. The BAG technique potentially decreases the need for gastropexy anchors, and has been successfully performed using a single gastropexy [8].

This study detected a statistically significant increase in the number of patients who underwent BAG placement for ventilator dependence, while less patients with head and neck malignancy received this dilation technique. This is likely due to the increased utilization of this technique amid the COVID-19 pandemic, and a decrease in the number of non-emergent procedures performed during this time frame.

There was no difference detected between procedure success rates or overall adverse event occurrence rate, suggesting the BAG method is not inferior to the serial dilation method for procedural safety. No major adverse events (SIR grade 3 AE or higher) occurred in the BAG group, and all infections occurred in the serial dilation group, a trend which was corroborated by prior studies [6]. This may be a consequence of the extra transcutaneous passes required when inserting multiple dilators to size up the tract. Depending on the final size of the gastrostomy tube, this may result in an additional 3 or 4 transcutaneous passes during the procedure. Numerous microorganisms are known to colonize the stomach, and performing multiple passes into the stomach lumen through a fresh skin wound may translocate microorganisms and increase the risk of infection [10]. Inserting multiple stiffened dilators into the stomach percutaneously also increases the risk of iatrogenic injury, such as inadvertent bowel perforation or t-fastener displacement.

Material costs were found to be higher in the BAG placement technique when compared to the serial dilation method. These costs are primarily driven by the price of the angioplasty balloon itself, which is being used off-label. Despite the increase in cost, the BAG technique offers reduced procedure times which may increase room turnover rate and productivity. These factors should be weighed carefully by the performing department to determine the most cost-effective solution.

Overall, the BAG technique of placing gastrostomy tubes is a safe and effective alternative to serial dilation with associated reduction in procedure times, radiation exposure, and decreased all-cause tube failure rates. These benefits are incurred with an increase in materials cost. These factors should be carefully considered when selecting a gastrostomy tube placement technique for optimal resource utilization and cost-effectiveness.

## Electronic Supplementary Material

Below is the link to the electronic supplementary material.

Visual abstract

## Data Availability

Data generated by the research are not openly available to protect the identities and healthcare privacy of the individual patients involved in the study. Generalized data may be available upon reasonable request to the corresponding author.
