# Correction to: Balloon assisted gastrostomy tube placement

**DOI:** 10.1007/s00261-025-05082-9

**Published:** 2025-08-27

**Authors:** Norbert Kuc, Ariel Felman, Ilan Small, Jacob Cynamon, Arash Gohari

**Affiliations:** 1 Montefiore Medical Center, The Bronx, USA; 2 Albert Einstein College of Medicine, The Bronx, USA; 3 Columbia University Irving Medical Center, New York, USA; 4 St. John’s Riverside Hospital, Yonkers, USA

**Correction to: Abdominal Radiology** 10.1007/s00261-025-04962-4

The original version of this article contained an error in one of the co-author’s family name. The co-author name should read as “Ariel Felman” instead of “Ariel Feldman”.

The original article has been corrected.

