# Supplementary material for: Balloon assisted gastrostomy tube placement
Source: Abdom Radiol (NY). 2025 Apr 30;50(11):5560–6. doi: 10.1007/s00261-025-04962-4 (PMC12568815; doi:10.1007/s00261-025-04962-4)
Supplement: Supplementary file 1 — Visual abstract [file 261_2025_4962_MOESM1_ESM.pptx]

## Slide 1
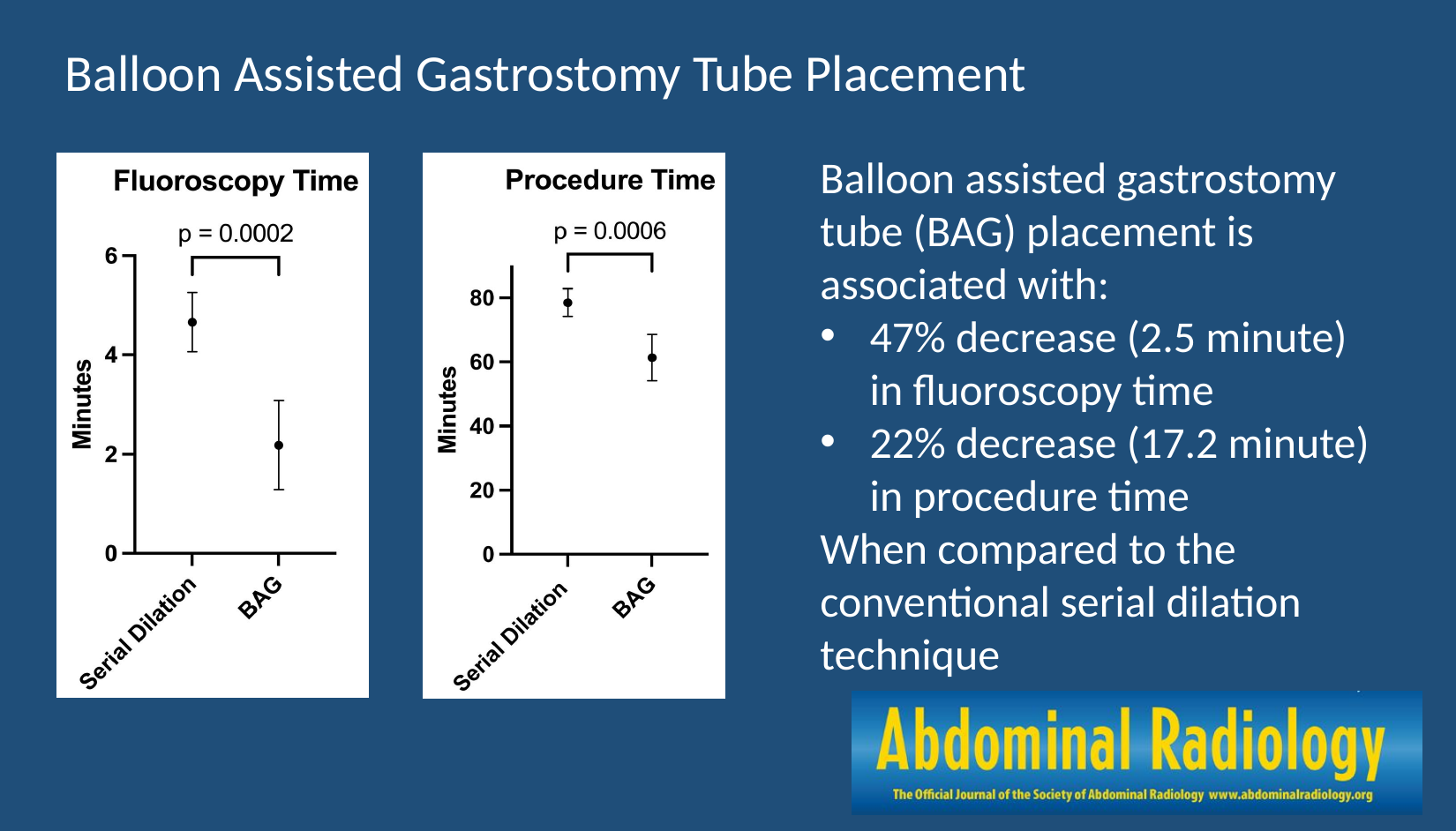

Balloon Assisted Gastrostomy Tube Placement
Balloon assisted gastrostomy tube (BAG) placement is associated with:
47% decrease (2.5 minute) in fluoroscopy time
22% decrease (17.2 minute) in procedure time
When compared to the conventional serial dilation technique
